# Severe neonatal cholestasis in *HNF1β* deficiency: a case report and literature review

**DOI:** 10.3389/fped.2025.1562573

**Published:** 2025-04-04

**Authors:** Chiara Gagliano, Olga Burattini, Luigi Paradisi, Sarah Recchione, Lucia Santoro, Laura Caponi, Annamaria Ciaschini, Maria Elena Lionetti, Simona Gatti

**Affiliations:** ^1^ Department of Pediatrics, University Polytechnic of Marche, Ancona, Italy; ^2^ Laboratory of Medical Genetics, Azienda Ospedaliero Universitaria Delle Marche, Ancona, Italy

**Keywords:** neonatal cholestasis, liver disease, congenital hypopituitarism, pituitary stalk interruption syndrome, 17q12 deletion, *HNF1β*

## Abstract

Neonatal cholestasis can be caused by several conditions, with biliary atresia being the major cause. Genetic and endocrinological etiologies represent other possibilities, with most of them requiring a rapid diagnosis and a specific treatment. We describe a neonatal case of severe cholestasis with low gamma glutamyl transferase in a child presenting with multiple abnormalities, including pituitary stalk interruption syndrome and consequent hypopituitarism. The cholestasis was rapidly resolved with hormone therapy. Genetic analysis showed a *de novo* 17q chromosome deletion, including the *HNF1β* gene implicated in liver damage, and this was considered causative of the complex clinical phenotype. Our case highlights the relationship between congenital hypopituitarism and *HNF1β* gene deletion in 17q12 deletion syndrome as a severe neonatal cholestasis etiology, emphasizing the need to be especially vigilant in cases with associated hypoglycemia. Prompt endocrine evaluation and genetic testing are crucial in neonatal cholestasis to start targeted therapy and long-term monitoring, which could mitigate serious complications.

## Introduction

Neonatal cholestasis (NC), characterized by conjugated hyperbilirubinemia, is not a benign condition, and the severity of its underlying causes should be promptly investigated (1). Biliary atresia (BA), occurring in 35%–41% of cases, is the major cause, but many other surgical and medical conditions (some potentially treatable) should be quickly excluded, with metabolic and endocrinological disorders representing up to 7% of cases (1). Endocrine diseases underlying NC are associated with significant morbidity and mortality risk due to severe hypoglycemia, acute adrenal insufficiency, and secondary hypothyroidism and require rapid hormone replacement treatment. Many of these conditions, manifesting soon after birth, are the result of a congenital pituitary malformation and/or are related to a specific genetic defect. Pituitary stalk interruption syndrome (PSIS), which is overall a very rare malformation with a reported incidence of 0.5/100,000 (2), is one the most frequent radiological presentations of congenital hypopituitarism (CH). Recently, an increasing proportion of neonatal/infantile cholestasis, previously defined as “idiopathic neonatal hepatitis,” has been recognized as a monogenic liver disorder. Genetic investigations are currently recommended early after the exclusion of BA in the workup of NC, in parallel with a metabolic workup (3). We describe the workup and follow-up of an infantile case of severe cholestasis in a patient with syndromic features (including hypopituitarism secondary to PSIS) and a final diagnosis of 17q12 deletion syndrome (including the *HNF1β* gene). While adult liver disease has been well described as a consequence of a *HNF1β* gene mutation/deletion, NC has rarely been reported and never in association with PSIS. This novel case is compared with reported cases of NC and *HNF1β* gene mutations.

## Case description

**Figure 1:**
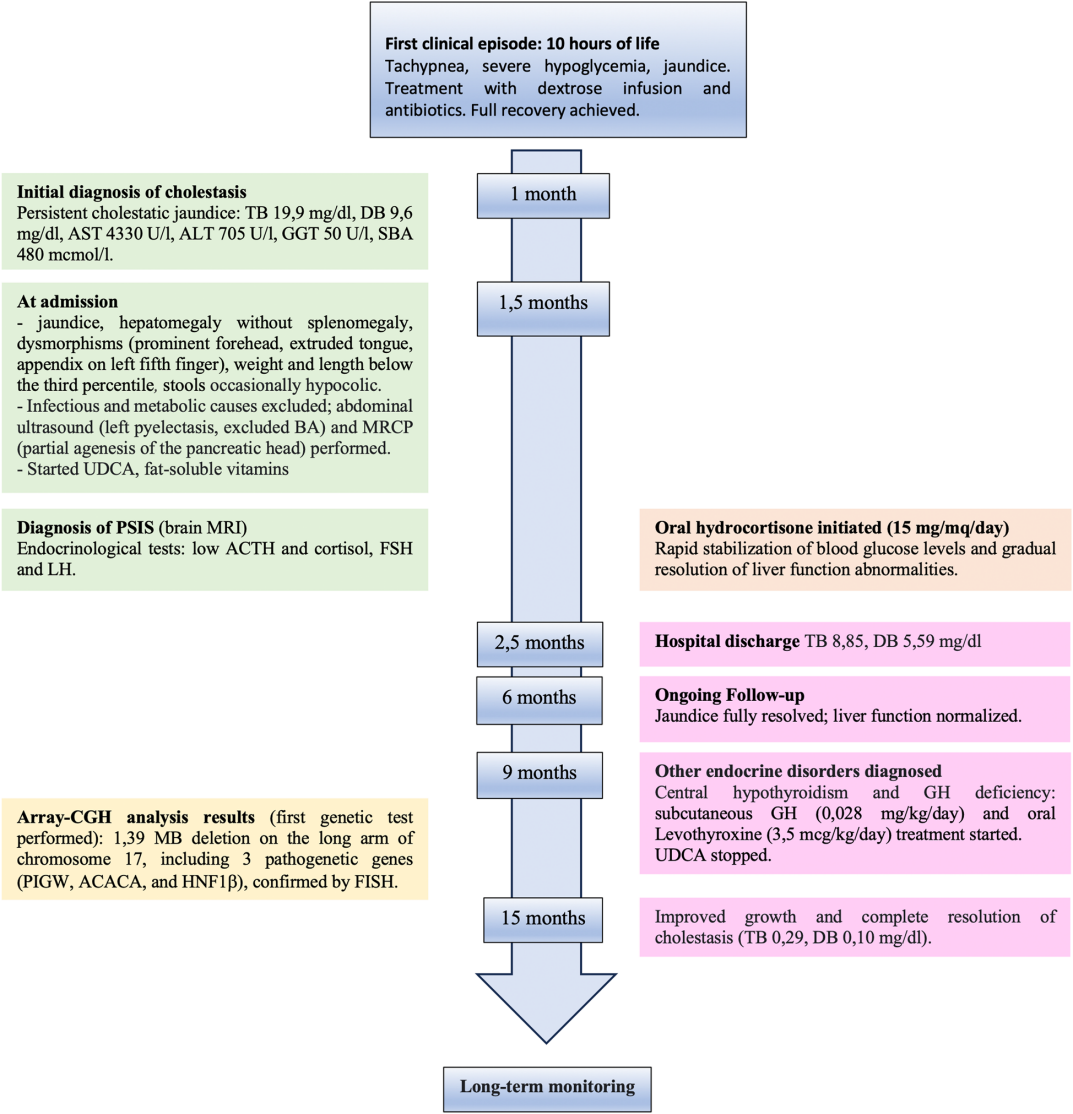
Timeline with chronological data and events from first clinical episode of severe hypoglycemia and hyperbilirubinemia to complete resolution after hormone replacement treatment. TB, total bilirubin; DT, direct bilirubin; AST, aspartate aminotransferase; ALT, alanine aminotransferase; GGT, gamma glutamyl transferase; SBA, serum bile acid; BA, biliary atresia; MRCP, magnetic resonance cholangiopancreatography; UDCA, ursodeoxycholic acid; PSIS, pituitary stalk interruption syndrome; MRI, magnetic resonance imaging.

A 43-day-old boy born at term from non-consanguineous parents (birth weight of 3,080 g, 11th percentile; birth length of 49 cm, 13th percentile) was referred to our attention for persistent cholestatic jaundice (for the timeline, see Figure 1). His family history included autoimmune thyroid disorder in the mother. At 10 h of life, he developed tachypnea, severe hypoglycemia, and jaundice, requiring a dextrose infusion and antibiotics due to a suspicion of sepsis, with full recovery. At 1 month of age, blood tests revealed cholestatic liver disease [total bilirubin: 19.9 mg/dl, direct bilirubin: 9.6 mg/dl, aspartate aminotransferase (AST): 4,330 U/L, alanine aminotransferase (ALT) 705 U/L, gamma glutamyl transferase (GGT): 50 U/L, serum bile acids: 480 μmol/L]. There was no history of vomiting, diarrhea, or acholic stools. The baby was transferred to our Pediatric Gastroenterology Department for further management. At admission, he had icteric skin and sclera, an enlarged liver (2 cm below the right costal margin) without splenomegaly, and normal cardiac and neurological examinations. Minor dysmorphisms, including a prominent forehead, extruded tongue, and an appendix on his left fifth finger, were observed. His weight and length were below the third percentile. His stools appeared normochromic but were occasionally hypocolic. Cholestatic jaundice was confirmed with mild derangement of liver synthetic function [hypoalbuminemia: 2.6 g/dl, mild coagulopathy with international normalized ratio (INR) 1.56] and ammonia values at the upper limit for his age. Serum GGT levels were almost within normal limits (55 U/L, normal 0–73 U/L). Plasma glucose level was frequently at the lower limit of normal (nadir: 36 mg/dl).

## Diagnostic assessment, therapeutic intervention, follow-up, and outcomes

**Figure 2:**
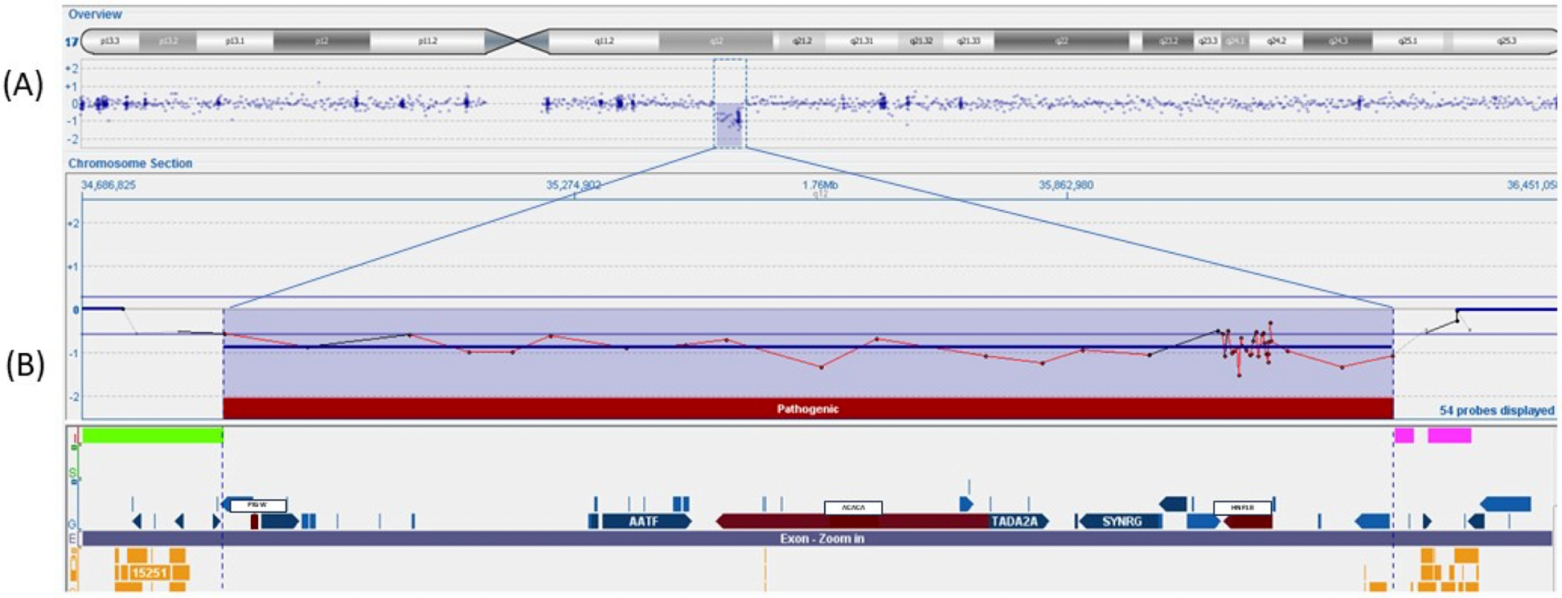
Array-CGH 400K analysis of the patient in which a deletion [arr(hg19) 17q12(34856305_36248926)x1] of 1.39 Mb in the region17q12 was identified. (**A**) Overview of the genes (refSeq) included in this deleted region. (**B**) The region contains 13 genes, and the three pathogenetic genes (*PIGW, ACACA,* and *HNF1β*) are marked in red.

The principal infective causes (viral hepatitis, toxoplasmosis, parasites, and bacterial infections) and metabolic liver diseases (normal urine organic acids, alpha1-antitrypsin, galactosemia, plasmatic amino acids profile) were quickly excluded. An abdominal ultrasound demonstrated left pyelectasis, a distended edematous gallbladder, and hepatomegaly with hyperechoic parenchyma, with normal-appearing bile ducts. The absence of acholic stools and the low levels of GGT did not suggest the possibility of a biliary atresia. Magnetic resonance cholangiopancreatography (MRCP) showed partial agenesis of the pancreatic head; these findings were not considered explanatory of the laboratory abnormalities. The endocrinological workup revealed low cortisol and adrenocorticotropic hormone (ACTH) levels (0.7 μg/dl and 2 pg/ml, respectively) with undetectable luteinizing hormone (LH) and follicle-stimulating hormone (FSH) (LH <0.2 mU/ml, FSH <0.2 mU/ml). Thyroid function tests and growth hormone (GH) levels were within the norms. Prolactin values were elevated (45.3 ng/ml). A head magnetic resonance imaging (MRI) scan demonstrated an absent pituitary stalk and an ectopic posterior pituitary, compatible with PSIS. During hospitalization, IV fluids, oral ursodeoxycholic acid, and fat-soluble vitamins were started; albumin and packed red blood cells were transfused. Oral hydrocortisone (15 mg/mq/day) was initiated, with a rapid improvement in the patient's general clinical conditions, a quick stabilization of blood glucose levels, and gradual improvement of liver function tests. At hospital discharge (2.5 months of age), total and direct bilirubin were 8.85 and 5.59 mg/dl, respectively. At 6 months, the jaundice had fully disappeared, and clinical and biological parameters associated with cholestasis were resolved. At 9 months, his weight and length remained below the 3rd percentile. Repeated endocrinological tests showed central hypothyroidism [thyroid-stimulating hormone (TSH): 0.460 μU/ml; T4: 0.87 ng/dl] and GH deficiency [GH: 0.92 ng/ml, insulin-like growth factor 1 (IGF-1) <15 μg/L]; therefore, subcutaneous GH (0.028 mg/kg/day) and oral levothyroxine (3.5 μg/kg/day) were started. Considering the presence of multiple malformations, array-comparative genomic hybridization (aCGH) analysis was performed as the initial genetic test, and a 1.39 MB deletion was detected on the long arm of chromosome 17 (17q12), containing the *HNF1β* gene (Figure 2). The deletion was confirmed by fluorescence *in situ* hybridization (FISH) and was not found in the parents; therefore, it was considered causative of the clinical phenotype. At 15 months of age, the child showed improved growth (in the 3rd percentile) and a complete clinical and laboratory resolution of cholestasis was observed (Table 1). Repeated abdominal ultrasounds confirmed the partial agenesis of the pancreatic head and left pyelectasis; pancreatic elastase was within the normal range.

**Table 1:** Results of liver function tests during hospital stay and at follow-ups.

Age (months)	Event	AST n.v. <40 U/L	ALT n.v. < U/L	GGT n.v. <55 U/L	Biliary acids n.v. <7.5 mcmol/L	Total bilirubin n.v. <1.5 mg/dl	Direct bilirubin n.v. <0.5 mg/dl
1.5	Hospital admission (start UDCA)	>1,000	542	54	643.95	18.93	12.12
2	Before starting hydrocortisone	355	77	73	530.7	13.22	8.29
2.5	Hospital discharge	254	139	79	387	8.85	5.59
3	Follow-up	327	486	107	157.5	3.9	2.82
5	Follow-up	87	153	82	4	0.3	0.16
9	Follow-up	41	20	18	14.02	0.34	0.1
15	Follow-up	HE	47	11	6.70	0.29	0.1

AST, aspartate aminotransferase; ALT, alanine aminotransferase; GGT, gamma glutamyl transferase; n.v., normal values; UDCA, ursodeoxycholic acid; HE, hemolyzed.

## Discussion

We have reported a case of severe NC caused by *HNF1β* deficiency related to CH, with a favorable outcome after hormone replacement therapy. This case report is the first to describe the combination of these two clinical entities (CH and NC) in the context of 17q12 deletion syndrome. Only two previous reports have described the association of CH with chromosome 17q deletion (4, 5), and a total of seven cases, apart from ours, have reported NC with this genetic defect (related to the deletion of the *HNF1β* gene) (Table 2) (6–12).

**Table 2:** Clinical and biochemical features of patients with *HNF1β* gene mutation-related disease and neonatal cholestasis.

Ref.	GW/BW g (SDS)	Age at diagnosis	Total bilirubin (conjugated) (mg/dl)/GGT (U/L)	Liver biopsy	Malformations/other comorbidities	Genetic analysis	Follow-up duration	Therapy	Outcome
Present report	41 + 1/3,080 (−1.13)	1 month of life	18.93 (12.12)/54	Not performed	Minor dysmorphisms, left kidney pyelectasis, hepatomegaly, partial agenesis of pancreatic head, hypopituitarism, hypoalbuminemia, hypoglycemia, anemia	1.39 Mb deletion including *HFN1B, PIGW, ACACA* genes, in 17q12 chr (*de novo*)	15 months	Hydrocortisone, GH, levothyroxine, fat-soluble vitamins, intravenous albumin, UDCA, blood transfusions	Gradual cholestasis resolution, growth in 3rd percentile
Raile et al., 2009 (case 1)	35/1,780 (−2.2)	First weeks of life	NA (elevated AST, ALT, GGT)	PILBD	Cystic kidney dysplasia, hydronephrosis due to urethral stenosis, chronic renal insufficiency, diabetes requiring insulin at 13 years, pancreatic hypoplasia with **progressive** exocrine pancreatic failure, inguinal hernia, abdominal testis, joint contractions, microcephaly, hypercholesterolemia, hypertriglyceridemia	Deletion in exons 1–9 of *HNF1β* (*de novo*)	18 years	Insulin therapy	Cholestasis resolution at 1 year follow-up with a persistent mild AST/ALT alteration, delayed psychomotor development, mental retardation, normal puberty, poor growth (final height −6.7 SDS)
Kitanaka et al., 2004	39/2,390 (−2.26)	1–2 months of life	NA (7.8)/NA	PILBD, marked cholestasis	Multiple bilateral renal cysts, mild chronic renal insufficiency, transient hypercholesterolemia, diabetes requiring insulin therapy at 13 years	Missense mutation in exon 2 of *HFN1B* gene, (c.457C>A, p.H135N, *de novo* or paternal)	13 years	Insulin therapy	Cholestasis resolution at 9 months with a persistent mild AST/ALT alteration, mild cognitive impairment
Beckers et al., 2007	37/1,520 (−4.24)	2 months	4.3 (3.5)/213	PILBD, severe biliary stasis, slight periportal fibrosis	Typical facies, bilateral posterior embryotoxon, recurrent cholangitis, hypertriglyceridemia, agenesis of left kidney, multiple renal cortical cysts with renal failure at 19 months, atrophic pancreas with progressive exocrine deficiency, non-autoimmune diabetes at 5 years	Deletion and insertion in exon 2 of *HFN1B* (*de novo*)	18 years	UDCA, insulin therapy, pancreatic enzymes substitution	Resolved cholestatic jaundice at 1 year (hepatic enzymes fluctuating), poor growth (final height −1.86 SDS), endocrine and exocrine pancreatic insufficiency
De Leusse et al., 2018	35 + 4/NA	NA (early life)	NA	Multinodular hepatic tumor (explanted liver)	Hepatocellular carcinoma (with no vascular invasion)	Deletion of chromosome 17	1 year	Liver transplant	NA
Kotalova et al., 2015	38/2,360 (−2.02)	First week of life (confirmed at 30 days with ERCP)	6.1 (1.87)/1,398	PILBD, severe biliary stasis, marked periportal fibrosis	Multiple bilateral cortical renal cysts, pancreatic hypoplasia, multiple cystic dysplasia of the left hepatic lobe, anemia	1,698 kb deletion including *HNF1β* (*de novo*)	2 years	Portoenterostomy sec. Kasai	Persistent liver dysfunction, growth in 3rd percentile, normal neurological development
Pinon et al., 2019	38/2,600 (−1.27)	5 weeks of life	11.95 (6.69) 221	PILBD, biliary stasis	Multiple bilateral cortical renal cysts, chronic renal failure, pancreatic exocrine disorder without hypoplasia, mild hyperparathyroidism, anemia	Missense mutation in exon 4 of *HFN1B* gene (c.827G>A, p.R276Q, *de novo*)	18 months	Fat-soluble vitamins, hydrolyzed protein milk, EPO	Persistent cholestasis and pruritus, normalized stools, mild chronic renal failure, normal neurological development
Weckwerth et al., 2021	NA	6 weeks of life	NA	PILBD, zone 3 canalicular cholestasis, no significant portal fibrosis	Bilateral renal cysts with normal renal function, posterior embryotoxon, severe metabolic bone disease with long bone fractures, vitamin D deficiency despite treatment, decompensated cirrhosis (jaundice, coagulopathy, peripheral edema, and hepatic encephalopathy) at age 14	c.884G>A mutation in *HFN1B* gene	16 years	Liver transplant at 15 years, insulin therapy	Good functioning of transplanted liver, insulin-dependent diabetes mellitus post-transplant on immunosuppressive therapy

GW, gestational weeks; BW, birth weight; PILBD, paucity of interlobular bile ducts; SDS, standard deviation score; ALT, alanine aminotransferase; AST, aspartate aminotransferase; GGT, gamma glutamyl transferase; NA, not available, EPO; erythropoietin, UDCA, ursodeoxycholic acid; ERCP, endoscopic retrograde cholangiopancreatography.

Our clinical report underlines the importance of evaluating endocrine disorders as one of the etiologies of NC. In a systematic review of 1,692 infants with NC, endocrinological disorders represented less than 2% of cases (1). PSIS is a rare developmental pituitary defect, generally presenting at birth or in the first months of life with hypoglycemia, failure to thrive, jaundice, cryptorchidism, and seizures (2). Cholestasis is frequently reported in patients with PSIS, with a prevalence ranging from 6% to 42% in different studies (13). It has been suggested that a decreased plasma cortisol level might be the precipitating factor for cholestasis, even if an effective role of GH and/or the TSH axis cannot be excluded (14). Both the type of liver derangement in our patient, characterized by very high aminotransferases and bilirubin with almost normal GGT, and the timing of resolution were in line with data from the literature, describing normal/low GGT in up to 43%–57% of cholestasis associated with CH, and resolution between 2 and 9 months after replacement therapy initiation (15). We confirm, therefore, that hormone replacement therapy is the basis of treatment for liver cholestatic associated with CH.

CH has been related to known genetic causes in only a small proportion of cases with non-syndromic and syndromic etiologies (16). PSIS, characterized by a triad of a thin/absent pituitary stalk, ectopic posterior pituitary, and aplasia/hypoplasia of the anterior pituitary, is a radiological diagnosis contributing to a high proportion (11.8%–34.2%) of CH cases (17).

The exact etiology of PSIS remains unclear with elusive underlying mechanisms. Many theories were initially proposed, such as adverse perinatal events affecting the hypothalamic-pituitar*y* axis (4). Recent findings suggest genetic origins involving pituitary development, neural development, axonal migration, and other important cellular processes that may act as predisposing factors, combined with environmental effects (2, 18).

CH patients with consistent/syndromic features, family history, or consanguineous parents are candidates for genetic testing, typically through next-generation sequencing (NGS), although some mutations may be detectable on array-CGH. In accordance with a recent study (18), PSIS should be considered part of the phenotypic spectrum of many genetic syndromes; therefore, an exome sequencing approach can more accurately characterize the genetic basis, compared to single gene analysis, retrieving genetic mutations in only 5% of PSIS cases (16).

Our initial genetic approach was based on array-CGH, which showed a *de novo* 17q12 deletion. The frequency of 17q12 deletion syndromes is estimated to be approximately 1:20.000. The deletion of 17q12 includes several genes, and among these, the *HNF1β* gene [related to maturity-onset diabetes of the young type 5 (MODY5)] is the best characterized. As in our case report showing left pyelectasis, urinary tract abnormalities have been detected in more than 50% of patients with *HNF1β* deficiency, with a significant heterogeneity. Neurodevelopmental disorders can occur, especially in large deletions as the one reported in the present case (19, 20). Other typical features include endocrinological disorders, with 25%–50% of patients having MODY5 and hyperparathyroidism; hypomagnesemia and hyperuricemia have also been reported (20). Pancreatic atrophy develops in up to 30% of cases, with the dorsal pancreas generally being more involved (20). Our patient showed early radiological evidence of pancreatic involvement (limited to the pancreatic head) with no signs of pancreatic insufficiency or dysfunction, suggesting the importance of a long-term endocrine follow-up and strict monitoring of pancreatic insufficiency. Mutations or deletions of the *HNF1β* gene have been described in correlation with different hepatic phenotypes, including NC, adult-onset cholestasis, non-cholestatic liver disease (6–12, 20–22), and, more recently, pediatric hepatocellular carcinoma (9, 23). The cholestasis resolved in all the described neonatal cases, except for one patient who developed hepatocellular carcinoma (9) and one who required a liver transplantation (12). Although our patient fulfilled most of the criteria of 17q12 syndrome, some peculiar features need to be noted, including the association with PSIS, the consequent multiple hormone deficiencies, and the severity of liver impairment.

In the literature, only one report has identified 17q12 deletion (including *HNF1β* gene) as a pathogenetic copy number variation (CNV), assessed with aCGH, in a non-syndromic patient with PSIS and isolated growth hormone deficiency. There is no reported evidence of CH with or without PSIS in syndromic patients with 17q12 deletion (5).

Another study described an association between 17q21 deletion and PSIS with growth hormone deficiency (GHD) and gonadotropic deficiency but no corticotropic deficiency (4).

The degree of cholestasis was probably the consequence of different etiologies (the pituitary insufficiency and deletion of *HNF1β*). This is emphasized by the singular biochemical profile of our case with low GGT, which is not reported in *HNF1β* deficiency (24) but is typical in CH. In our workup of the cholestasis, considering the early and convincing diagnosis of CH, we decided not to perform a liver biopsy; therefore, we cannot completely exclude an underlying histological abnormality of the liver. All the reported neonatal cases showed a paucity of biliary ducts associated with marked cholestasis and variable periportal fibrosis (Table 2). All the cases, except one (9), had concomitant renal malformations (typically multiple renal cysts with a variable degree of chronic renal insufficiency). Similar to our case, pancreas agenesis or hypoplasia was reported in three cases. Four patients subsequently developed diabetes, requiring insulin therapy at 5 years, 13 years, and 15 years, respectively (7, 8, 12). Our patient did not show developmental delay at the last follow-up, while a mild cognitive impairment was found in two patients at their last follow-up (13 and 18 years) (6, 7); however, no brain imaging was reported.

The genetic definition of our case was rapidly achieved by an array-CGH test. Although this was resolutive, we cannot exclude the presence of other genetic mutations that could be revealed by an exome sequencing study.

It is relevant to stress the importance of a periodic biochemical and liver ultrasound follow-up in our patient, even if there was a complete resolution of the cholestasis. In parallel, a high index of suspicion for the development of diabetes and exocrine pancreatic insufficiency should be constantly maintained considering the presence of pancreatic hypoplasia and the concomitant lifelong necessity of steroid replacement in a patient with this genetic predisposition.

In conclusion, our case suggests the possibility of a double etiology for neonatal cholestasis in patients with 17q12 deletion syndrome, including the deletion of the *HFN1β* gene and concomitant pituitary insufficiency. In the workup of NC, when there is a suspicion for *HNF1β* deficiency (particularly in patients with syndromic features and severe liver involvement) and NGS is negative, array-CGH or multiplex ligation-dependent probe amplification (MLPA) could be indicated.

## Data Availability

The original contributions presented in the study are included in the article/Supplementary Material, further inquiries can be directed to the corresponding author.
